# Artefactual depiction of predator–prey trophic linkages in global soils

**DOI:** 10.1038/s41598-021-03234-7

**Published:** 2021-12-13

**Authors:** Kris A. G. Wyckhuys, Ha Nguyen, Steven J. Fonte

**Affiliations:** 1 Institute of Plant Protection, China Academy of Agricultural Sciences, Beijing, China; 2 Fujian Agriculture and Forestry University, Fuzhou, China; 3 University of Queensland, Brisbane, Australia; 4 Chrysalis Consulting, Hanoi, Vietnam; 5 Center for Agricultural Research and Ecological Studies, Vietnam National University of Agriculture, Hanoi, Vietnam; 6 Department of Soil and Crop Sciences, Colorado State University, Fort Collins, CO USA

**Keywords:** Agroecology, Ecosystem services

## Abstract

Soil invertebrates contribute to multiple ecosystem services, including pest control, nutrient cycling, and soil structural regulation, yet trophic interactions that determine their diversity and activity in soils remain critically understudied. Here, we systematically review literature (1966–2020) on feeding habits of soil arthropods and macrofauna and summarize empirically studied predator–prey linkages across ecosystem types, geographies and taxa. Out of 522 unique predators and 372 prey organisms (constituting 1947 predator–prey linkages), the vast majority (> 75%) are only covered in a single study. We report a mean of just 3.0 ± 4.7 documented linkages per organism, with pronounced taxonomic biases. In general, model organisms and crop pests (generally Insecta) are well-studied, while important soil-dwelling predators, fungivores and detritivores (e.g., Collembola, Chilopoda and Malacostraca) remain largely ignored. We argue that broader food-web based research approaches, considering multiple linkages per organism and targeting neglected taxa, are needed to inform science-driven management of soil communities and associated ecosystem services.

## Introduction

Globally, the contribution of vertebrates and aboveground biota to ecosystem functioning and human well-being is relatively well-recognized, but the diversity, feeding patterns and ecosystem services provided by invertebrates and soil-dwelling biota are critically underappreciated^1,2^. Soils harbor a vast reservoir of biodiversity, with an estimated 25% of the Earth’s species, and also contribute immensely to the regulation of global biogeochemical cycles and the health and welfare of human society^3–5^. A myriad of soil biota act as critical biochemical, physical or ecological mediators of ecosystem function^6,7^, with invertebrate activities tied to ecosystem service bundles that comprise erosion control, nutrient cycling, carbon capture or water storage^8–10^. Yet, most soil-dwelling organisms remain overlooked and their scientific coverage experiences marked spatial and taxonomic biases^11,12^, with only 0.3% of sampling sites concurrently yielding biodiversity and function data^5^. In recent decades, soil ecological research has tended to focus on microbial communities^13^, over-emphasized the value of coarse-grained metrics e.g., richness of autotrophs^5,14^ and paid scant attention to the functional roles of soil-dwellers^15^. Also, due to the complex and opaque nature of the soil habitat, trophic interactions among organisms with diverse feeding habits cannot easily be elucidated and are rarely mapped at fine taxonomic grain^16^. Finally, soil invertebrate taxonomic knowledge is sorely incomplete with a respective 83%, 77% and 45% of the world’s Collembola, earthworms or mites yet undescribed^17^. Even in agroecosystems that have been managed for centuries, species-rich communities of mesofauna e.g., predatory mites, still await discovery and taxonomic description.

Processes such as herbivory, decomposition and nutrient cycling are regulated by higher-order consumers such as predators^18,19^. Soil biodiversity is thought to support multi-functionality^20,21^ and overall ecosystem stability^22,23^. Top-down control by soil-borne predators can provide insurance against biodiversity loss and other global change disruptions^24–26^. High-order consumers such as generalist predators further link food webs over space and time, coupling habitats and energy channels, and thereby shape population dynamics of resource species (e.g., herbivorous prey)^27,28^. Generalist predators equally connect belowground (BG) food webs with aboveground (AG) habitats, assuming a bridging role between both sub-systems similar to that of plants^29,30^. Yet, the ecosystem functions tied to these higher trophic levels within different functional domains, e.g., phyllosphere, rhizosphere, are rarely considered^5,31^. To date, there’s no comprehensive understanding of the prevalence, strength and direction of predator–prey trophic linkages in global soil food webs. Overall, while the diversity of several soil animals has been mapped at a global scale^11,32^, the overarching patterns and mechanistic basis of soil food web dynamics remain poorly understood^5,6^.

Aside from acting as integrators of distinct ecosystem compartments, generalist predators directly support biological control of crop-feeding pests, thereby mitigating pest-induced losses and underpinning agri-food production^33,34^. Predators exhibit inconsistent responses to non-crop habitat surrounding individual agricultural fields^35^. Hence, field-level features such as the relative disturbance regime, cropping sequence, vegetational complexity and soil health are expected to be key determinants of biological control^36,37^. Abundance, identity and quality of decomposer prey within the BG subsystem are of fundamental importance to biological control, while many predators do not benefit from habitat structural complexity per se^29,38,39^. Given that most crop protection studies consider AG and BG subsystems in isolation^40^, empirical insights and theoretical constructs are lacking to formulate effective soil-targeted interventions for field crops^41^. This lack of holistic, integrative perspectives hampers a proper identification of AG- or BG-level management targets for ecological engineering^42,43^. Instead, a plethora of pest-centric studies employing simplified (i.e., bi-trophic) frameworks have examined a suite of (single-factor) interventions e.g., addition of animal manure or alternative prey, to bolster biological control, routinely yielding serendipitous outcomes and management recommendations that are only valid under particular agroecological contexts or pest-crop systems^44^. We argue that a systematic mapping of consumer-resource linkages across AG-BG systems can lay the groundwork for more targeted manipulations of soil-dwelling biota and facilitate a science-driven ecological intensification of the world’s farming systems.

In this study, we systematically review the global scientific literature on soil-based predator–prey trophic interactions and gauge the extent of scientific attention to the associated ecosystem service of biological control. Based on an extensive literature screening, we consider studies that either empirically demonstrate (i.e., realized links) or confidently deduce (i.e., inferred links) soil-borne trophic linkages. The former set of studies relied upon visual observations, predator exclusion trials or feeding assays, while the latter deduced linkages through advanced methods such as stable isotope or fatty acid analysis. Literature queries aimed to capture trophic interactions for macrofauna (invertebrates > 2 mm in size) and arthropod mesofauna (i.e., mites and collembolans) in global soils, but omitted nematodes (which are regularly considered pathogens instead of predators^45^). Organisms were hereby termed ‘soil-dwellers’ when their life cycle either entirely or partially took part within or on the soil. For the above biota, we logged feeding processes with a broad suite of AG- or BG-taxa, including vertebrates that feed on macrofauna. Next, we mapped organismal links at varying taxonomic grain and partitioned the prevailing AG or BG taxa within consumer or resource guilds, including target or amplifiable prey^46^. Lastly, we plotted the degree of taxonomic mismatch between realized and inferred trophic links and enumerated taxa for which current scientific attention is not well-aligned with their functional importance. By thus diagnosing scientists’ portrayal of (soil) food web interactions, we identify critical knowledge gaps, enable improved trophic grouping of soil dwellers and aim to facilitate a scientifically-driven management of coupled AG-BG systems.

## Results

**Figure 1:**
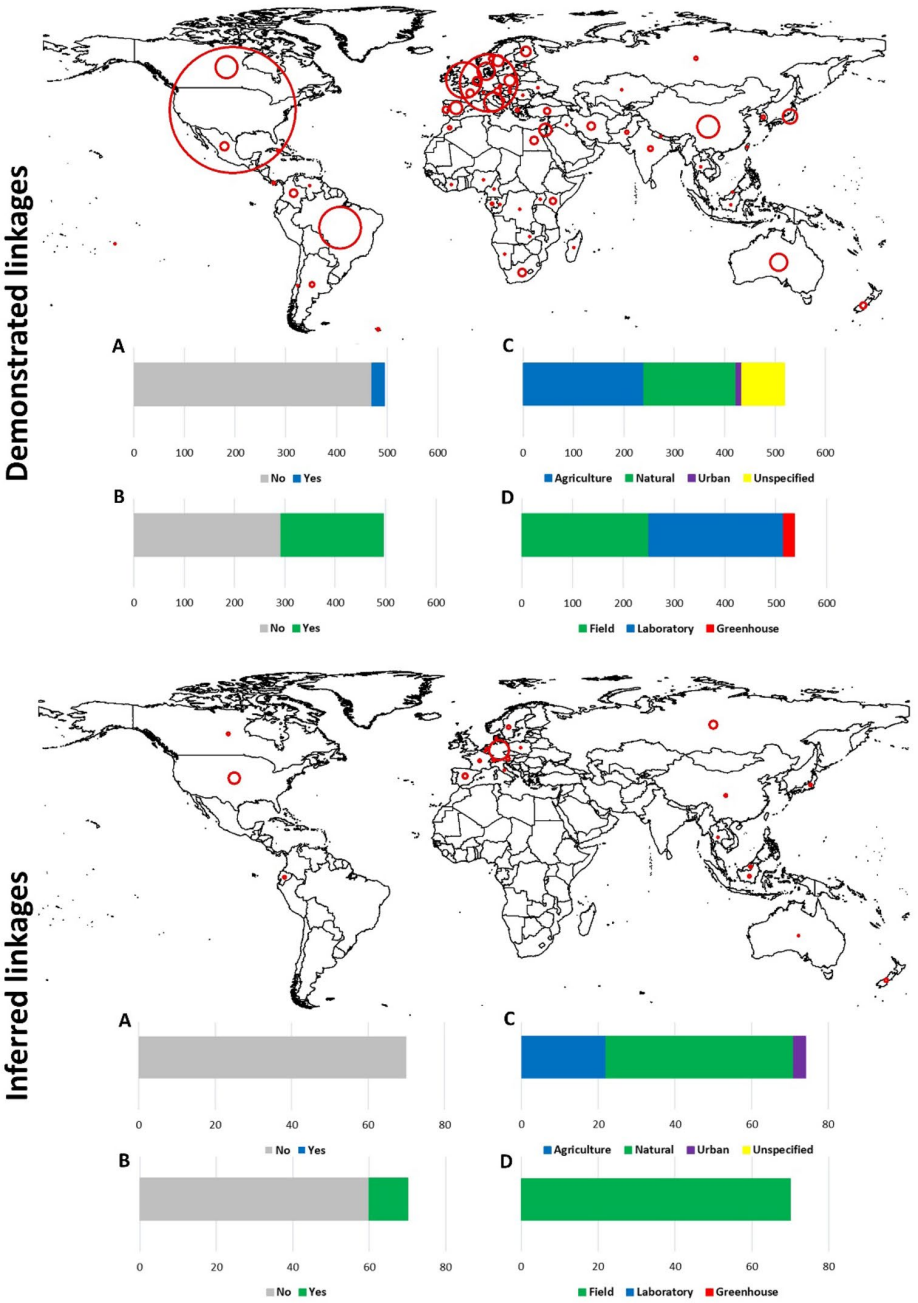
Geographical distribution and key features of scientific studies that either empirically demonstrated (upper panel) or inferred (lower panel) soil-borne trophic linkages. On either world map, red circle sizes are indicative of the number of studies per country. Below each map, bar charts show whether individual studies considered impacts on primary productivity (yes/no; **A**) or biological control (yes/no; **B**). Bar charts (**C**) and (**D**) cover the targeted habitat type (agriculture, natural, urban, unspecified) and the type of assay (field, laboratory, greenhouse). All bar charts depict the absolute number of publications, within a given sub-set of studies. Maps were created using ArcMap 10.6.1.

By querying the Web of Science Core Collection database (1900–2020), we captured literature records in which soil-dwelling invertebrates belonging to 36 different taxa (Supplementary Table 1) acted as either predators or prey items and logged the associated resource or consumer organisms (e.g., other invertebrates or vertebrates such as frogs, birds and lizards). The terms predator or consumer organism, and prey or resource organism are hereby used interchangeably. Out of 2208 unique literature records, 495 studies were selected in which trophic linkages were empirically demonstrated (i.e., realized links), while another 70 studies involved deduced linkages (i.e., inferred links). Within the former sub-set of studies, 21% of records originated from the US followed by Germany (10% records), Brazil (7%) and the UK (6%) (Fig. 1). Most studies (95%) did not assess impacts on primary productivity (i.e., crop yield or biomass) and 59% did not make any reference to the ecosystem service of biological control. Nearly half of the studies investigated farmland ecosystems and involved field assays. The most popular techniques to illuminate trophic linkages included behavioral observation (*n* = 160), (sentinel) prey consumption (145), predator life history assays (78) and predator exclusion/addition trials (71). Novel techniques such as enzyme-linked immunosorbent assays (ELISA) or molecular gut content analyses were used in 38 studies, while traditional approaches such as fecal analysis, digestive tract dissection or brood cell (or nest, web) content examination were employed in a respective 19, 36 and 23 cases. Trophic linkages were logged for 522 different consumer organisms and 372 resource items, identified either at the species or genus level. Among all consumers, a given organism featured in 1.5 ± 2.3 (x̄ ± SD) studies and 3.2 ± 4.8 (non-unique) linkages (2.4 ± 2.9 consumer links per study). Hence, each predator species was reported to consume an average of 3.2 different prey species. Importantly, vertebrates such as the curlew *Burhinus oedicnemus* L. (*n* = 1), coati *Nasua nasua* L. (1) or armored shrew *Scutisorex somereni* Thomas (1) and the crabronid wasp *Oxybelus analis* Cresson (1) exhibited most (18–20) realized links per study. Among all resource species, a given organism featured in 1.6 ± 2.0 studies and 2.8 ± 4.4 linkages (1.7 ± 1.6 resource links per study). The locust *Dociostaurus maroccanus* (Thunberg) (*n* = 1), midge *Dasineura brassicae* Winn. (1) and deer tick *Ixodes scapularis* Say (1) exhibited the highest number (10–12) of trophic linkages per study. Resource organisms regularly only completed part of their life cycle in the soil e.g., egg pods of *D. maroccanus* egg pods or the eggs, gravid females and larvae of *I. scapularis*. Over 95% of registered biota featured in three or less studies; only one trophic linkage was established for a respective 48% and 56% of consumer and resource organisms (Supplementary Fig. 2).

**Figure 2:**
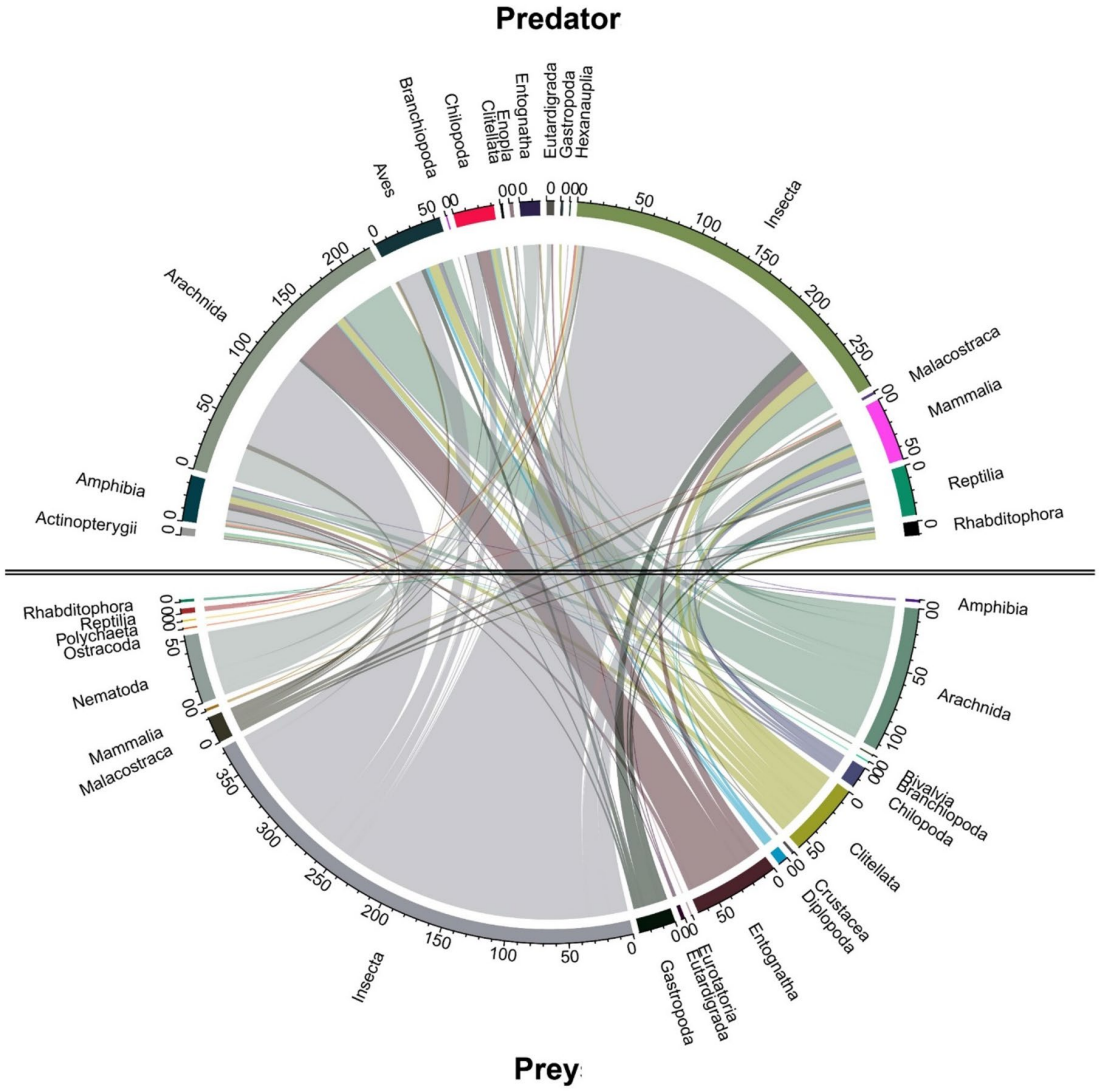
Chord diagram representing realized trophic linkages between consumer (predator; top) and resource (prey; bottom) items. Consumer-resource linkages are only visualized at the taxonomic hierarchy of class, and comprise numerous biota ranging from vertebrates (e.g., Aves, Mammalia, Amphibia), crustaceans (Malacostraca) to more common soil meso- and macrofauna. A total of 764 linkages are plotted, solely drawn from the empirical assessments of trophic linkages (*n* = 495 studies).

**Figure 3:**
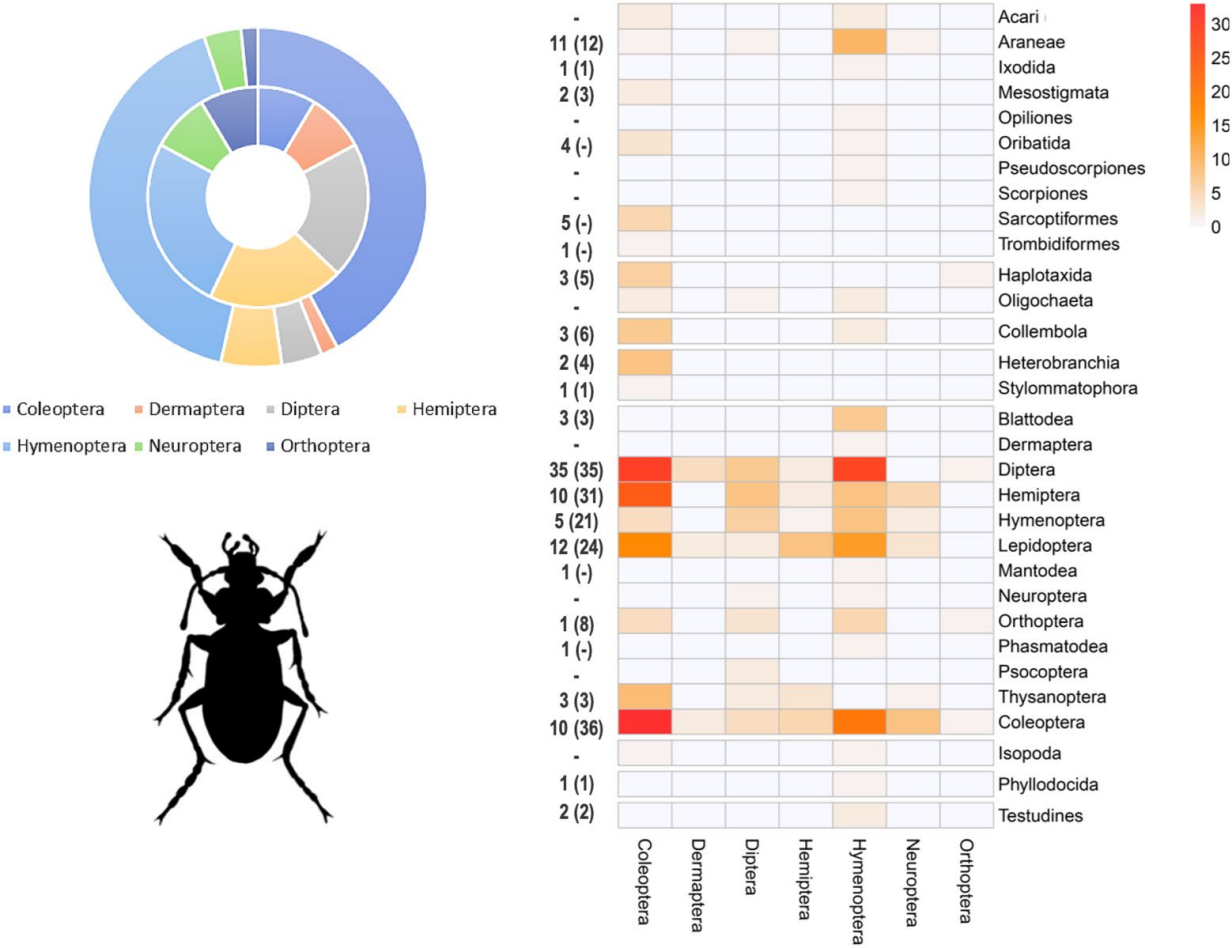
Order-level trophic linkages for Insecta consumer species. In the right panel, a heat map depicts the number of realized linkages between different consumer (column) and resource guilds (row). Numbers next to each row indicate the respective number of families and species (between brackets) within a given order of resource species. In the left panel, a donut chart shows the relative number of families (inner circle; total n = 35) and species (outer circle; total n = 310) within the 7 different orders of Insecta consumers.

**Figure 4:**
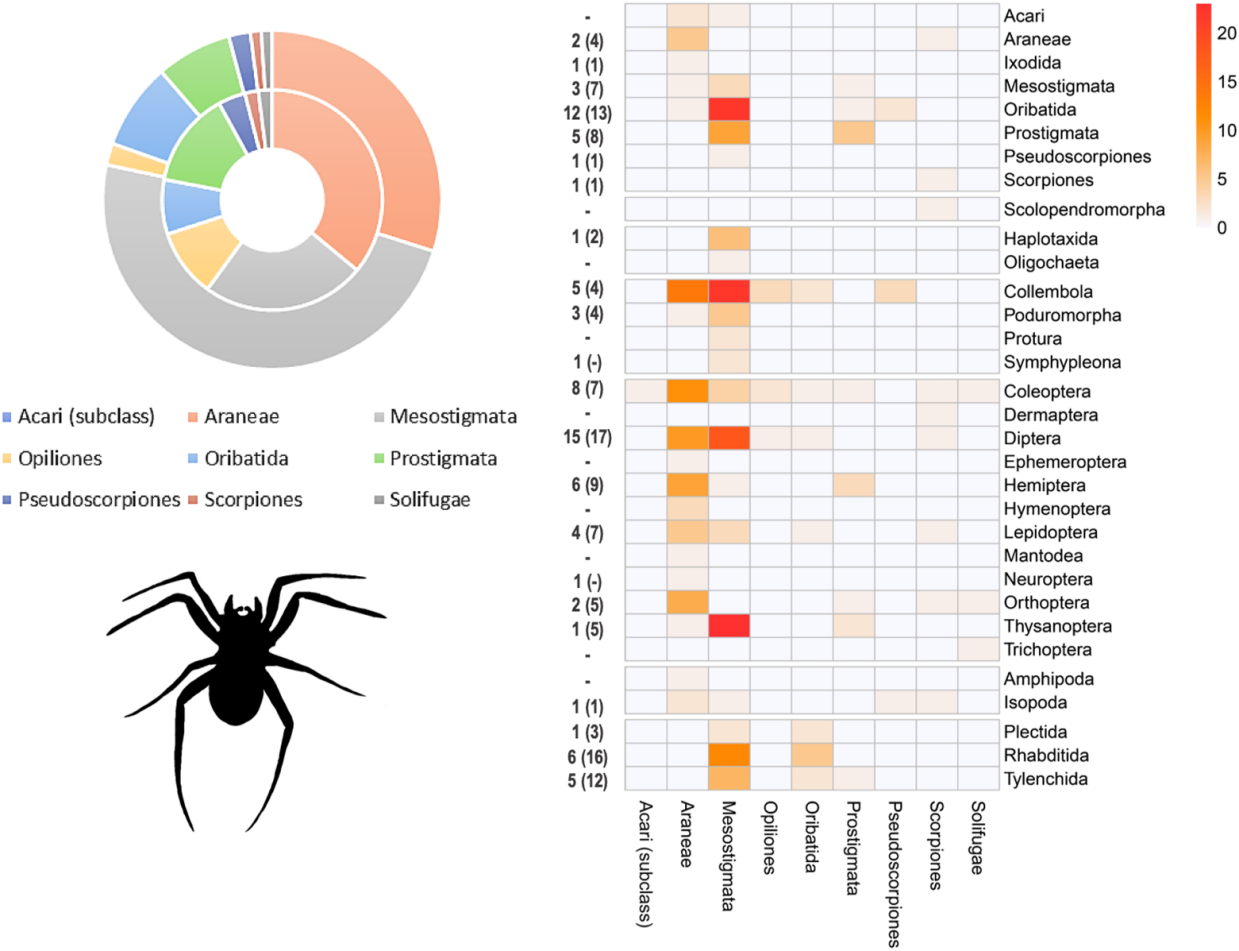
Order-level trophic linkages for Arachnida consumer species. In the right panel, a heat map depicts the number of realized linkages between different consumer (column) and resource guilds (row). Numbers next to each row indicate the respective number of families and species (between brackets) within a given order of resource species. In the left panel, a donut chart shows the relative number of families (inner circle; total *n* = 50) and species (outer circle; total *n* = 97) within the 8 different orders and one sub-class of Arachnida consumers.

From the 495 studies based on empirical linkages, a total of 1947 non-unique trophic linkages at variable taxonomic resolution were extracted. At the coarsest taxonomic hierarchy (i.e., class), a total of 763 linkages were drawn in which Insecta and Arachnida made up a respective 49% and 15% of resource items (Fig. 2), and 36% or 29% of consumer organisms. Other common resource items were Collembola, Protura or Diplura (*n* = 68) and Clitellata (58), while vertebrates such as Aves (54) and Mammalia (50) featured prominently among the consumer organisms. Among Insecta consumers, a total of 371 order-level trophic linkages were drawn with 31 distinct orders or sub-classes under eight animal classes (Fig. 3). These comprised mostly Coleoptera (43%) and Hymenoptera (32%) as consumer organisms, while Diptera (20%), Coleoptera (20%), Hemiptera (13%) and Hymenoptera (12%) ranked prominently among resource items. Surprisingly, common soil-dwellers such as Collembola (n = 9), Oribatid mites (4), Isopoda (2) or earthworms (sub-class Oligochaeta 5; Haplotaxida 7) were markedly underrepresented in the registered linkages with Insecta consumers. At the finest taxonomic grain, 310 species of Insecta belonging to 35 different families featured in the registered linkages. Among Arachnida consumers, a total of 277 unique order-level linkages were established with 32 distinct orders or sub-classes under seven animal classes as resource items (Fig. 4). These comprised largely Mesostigmata (52%) and Araneae (28%) as consumer organisms; Collembola (16%), Diptera (11%), Oribatid mites (9%) and Thysanoptera (9%) constituted common resource items. Nematodes (3 orders) featured as resource items in 11% linkages. At the finest taxonomic grain, 97 species of Arachnida belonging to 50 different families assumed a role as consumer organisms within trophic linkages. Cannibalism was recorded for just 7 out of the 1947 (non-unique) linkages, involving the rove beetle *Dalotia coriaria* (Kraatz), the mites *Gaeolaelaps aculeifer* (Canestrini) and *Stratiolaelaps scimitus* (Berlese), the lycosids *Tigrosa helluo* (Walckenaer) and *Pardosa milvina* (Hentz) and the scorpion *Mesobuthus gibbosus* (Brullé).

Consumer species that received most scientific attention included the fire ant *Solenopsis invicta* (Buren), commercially available predators such as *G. aculeifer* and *D. coriaria*, or charismatic macro-invertebrates such as *Pterostichus melanarius* Ill. and *Coccinella septempunctata* L. (Table 1). These species either preyed upon soil-dwelling organisms or on foliage feeders that completed part of their life cycle in the soil (e.g., thrips pupae). Other common prey items involved foliage feeders that dropped on the soil surface after being dislocated (e.g., *C. septempunctata* consuming dislodged aphids). For 7 out of the 10 most investigated consumer species, trophic linkages were established with both target prey (i.e., crop pests) and non-herbivore prey (e.g., amplifiable organisms such as bacterivores, fungivores^46^); prey targets variably belonged to AG and BG ecosystem compartments. The most studied resource species included prime agricultural pests such as the thrips *Frankliniella occidentalis* (Pergande) or the aphid *Rhopalosiphum padi* (L.), storage pests e.g., *Tyrophagus putrescentiae* (Schrank) and common laboratory organisms such as *Folsomia candida* Willem and *Tenebrio molitor* L. (Table 2). While for *F. occidentalis* or *R. padi* a broad suite of (soil- and foliage-dwelling) predators were identified and ample attention was given to biological control, such was not the case for all other resource organisms.

**Table 1:** Ten most investigated consumer species within empirical assessments of trophic linkages.

Species	Taxonomic classification	# studies	Links/study	Target/total prey species	AG/BG targets	Biological control
*Gaeolaelaps aculeifer*	Arachnida Mesostigmata	34	1.71	7/27	3/4	0.50
*Stratiolaelaps scimitus*	Arachnida Mesostigmata	28	1.92	15/26	5/10	0.89
*Pterostichus melanarius*	Insecta Coleoptera	20	1.70	11/18	9/2	0.50
*Harpalus rufipes*	Insecta Coleoptera	10	1.20	10/10	9/1	0.80
*Solenopsis invicta*	Insecta Hymenoptera	9	1.11	7/9	6/1	0.78
*Dalotia coriaria*	Insecta Coleoptera	9	2.00	7/11	2/5	1.00
*Nebria brevicollis*	Insecta Coleoptera	7	1.57	8/10	6/2	0.71
*Coccinella septempunctata*	Insecta Coleoptera	5	1.20	5/5	5/0	0.57
*Coleomegilla maculata*	Insecta Coleoptera	5	1.20	4/4	4/0	1.00
*Myrmeleon hyalinus*	Insecta Neuroptera	5	1.00	1/1	0/1	0.00

For each species, the total number of studies and the average number of trophic links per study is recorded. Among associated resource items (i.e., prey), the number of target species (Ferris et al., 2012) and their respective foraging habits (i.e., above-ground AG, below-ground BG) are logged. Records solely include consumers identified at the species level and resource items identified at the genus or species level. For each consumer species, the proportion of studies that dedicate explicit attention to biological control is indicated.

**Table 2:** Ten most investigated resource species within empirical assessments of trophic linkages.

Species	Taxonomic classification	# studies	Links/study	# predators	SD/FF	Biological control
Frankliniella occidentalis*	Insecta Thysanoptera	29	1.76	25	10/14	0.97
Rhopalosiphum padi*	Insecta Hemiptera	13	1.92	23	15/7	0.69
Tyrophagus putrescentiae*	Arachnida Sarcoptiformes	12	1.42	7	5/2	0.58
*Folsomia candida*	Entognatha Collembola	10	1.00	5	4/0	0.20
Tenebrio molitor*	Insecta Coleoptera	9	1.22	6	6/0	0.22
Deroceras reticulatum*	Gastropoda Heterobranchia	8	1.12	5	5/0	0.63
Aphis gossypii*	Insecta Hemiptera	7	1.14	7	0/7	0.57
Diabrotica virgifera*	Insecta Coleoptera	7	5.57	18	18/0	0.86
Musca domestica*	Insecta Diptera	7	1.00	6	5/0	0.29
Tetranychus urticae*	Arachnida Trombidiformes	7	1.14	6	0/6	0.71

For each species, the total number of studies, the average number of trophic links per study, the number of associated consumer items (i.e., predators) and their respective foraging habits (i.e., soil-dweller SD, foliage-forager FF) are logged. Records solely include resource items identified at the species level and consumers identified at the genus or species level. For each resource species, the proportion of studies that dedicate explicit attention to biological control is indicated. Target prey (e.g., herbivore, storage or nuisance pests) are indicated with an asterisk.

Within the subset of studies that did not empirically demonstrate linkages, 23% of the records originated from Germany, while the US and Russia yielded a respective 13% and 9% of records (Fig. 1). All studies disregarded primary productivity and as few as 14% studies considered biological control services. Seventy percent of studies investigated natural ecosystems (e.g., temperate broadleaf or pine forest) and all studies exclusively involved field assays. In 59 studies, researchers employed stable isotope analysis to infer trophic linkages, while a respective 11 and 5 studies relied upon metal bio-accumulation or fatty acid/lipid analysis. At the taxonomic hierarchy of order, 11 and 12 taxa featured within the respective upper (inferred predators) and lower trophic (i.e., inferred prey) levels (Supplementary Fig. 3). Within the upper trophic level, Arachnida (38%), Insecta (33%) and Chilopoda (17%) featured prominently among 114 unique order-level records (recorded per study). Conversely, within the lower trophic level, Insecta (28%), Collembola, Protura or Diplura (20%), Arachnida (15%) and Clitellata (13%) were well-represented among 131 unique order-level records. At a finer taxonomic grain, a total of 32 distinct orders or sub-classes and 81 families were logged within upper trophic levels, while 39 different orders or sub-classes and 84 families featured in the lower trophic level (Supplementary Fig. 3).

**Figure 5:**
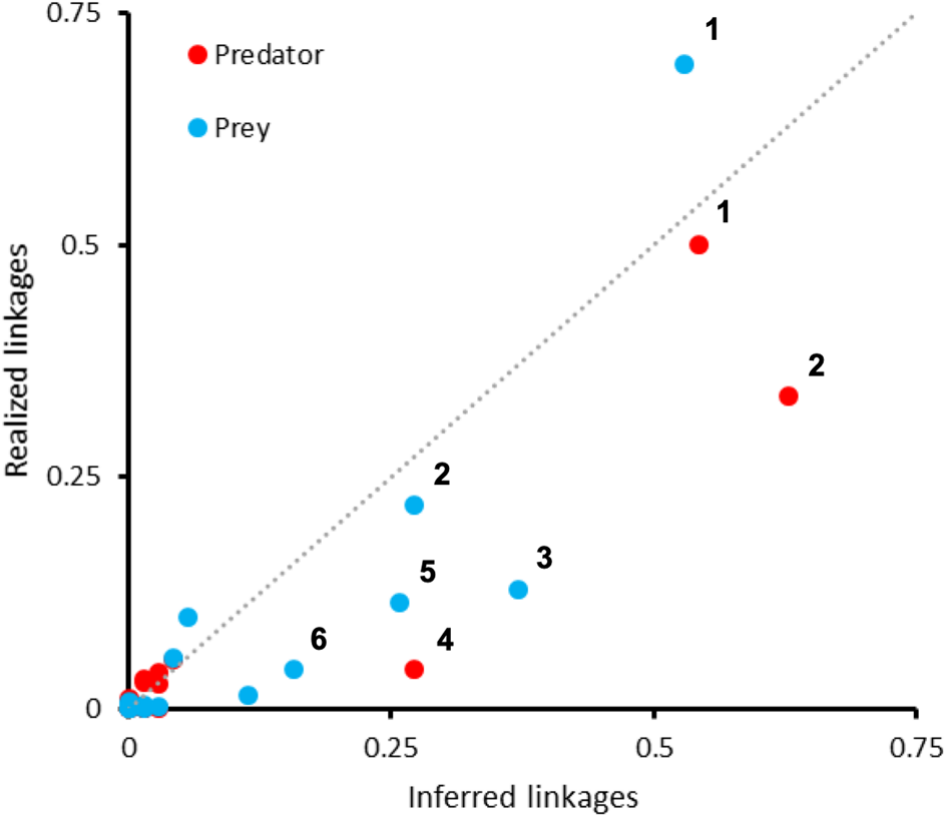
Extent of taxonomic mismatch between studies that either infer or empirically demonstrate trophic linkages. Axes reflect how 24 different classes of soil-dwelling animal biota proportionally feature with realized (Y axis) or inferred (X axis) linkages. For each taxon, the proportion of studies that infer its presence in either upper (i.e., predator) or lower (i.e., prey) trophic levels is plotted against the relative number of actual realized trophic linkages (i.e., the latter drawn from empirical assessments of consumer-resource interactions). Selected taxa are indicated: 1 Insecta; 2 Arachnida; 3 Entognatha; 4 Chilopoda; 5 Clitellata; 6 Malacostraca; with the majority of Enthognatha, Clitellata and Malacostraca being Collembola, earthworms and terrestrial Isopoda, respectively. The diagonal line mirrors equal extent of organismal coverage between both types of studies.

When contrasting the extent of organismal attention (class-level taxonomic hierarchy) between studies that either empirically derived or inferred trophic interactions, marked patterns are observed. Visualization of this data via scatter plot (Fig. 5) indicates that the most prominent taxa, Insecta consumers and resources are moderately overrepresented within realized (i.e., empirically demonstrated) linkages. Similarly, Arachnida or Chilopoda consumers and Entognatha, Clitellata, Diplopoda and Malacostraca resource items are considerably underrepresented in empirical assessments of trophic linkages. Among taxa that receive lesser amounts of scientific attention, Nematoda or Gastropoda resource items and Aves, Mammalia, Reptilia or Entognatha consumers disproportionately feature within realized links. Further, there’s a distinct overrepresentation of Chilopoda resources within inferred trophic links (or a critical underrepresentation in realized links).

## Discussion

Soil biodiversity provides multiple ecosystem services that are often irreplaceable by external inputs and threatened by intensive farming^47^. Thriving soil communities can enhance nutrient cycling and water dynamics, help to sustainably increase global food production^8,48^, and bolster overall resilience of terrestrial ecosystems^49^. Notwithstanding the societal importance of healthy soils, relatively little is known about inter-organismal linkages within soil food webs and the extent to which those anchor aboveground ecosystem compartments^31,37,50^. Lifting the lid on the soil “black box”^1,12,17,51^, we show how predator–prey functional assessments are chiefly made in North America and western Europe. Also, among the 894 investigated species, most receive superficial scientific attention with 81% of consumer and 76% of resource species featuring in only a single study. Within the orders of Insecta or Arachnida, conspicuous ground beetles (Carabidae) or predatory mites (Mesostigmata) act within 43% and 52% class-level trophic links. Associated prey items regularly belong to other Insecta classes and often constitute target prey (i.e., herbivorous crop pests^46^). Popular consumer species include (commercial) biological control agents and charismatic macro-invertebrates, while (aboveground) agricultural herbivores, storage pests and laboratory model organisms such as the collembolan *F. candida* feature prominently among studied resource items. Given the important taxonomic biases in predation studies, ecologists rely upon a simplified representation of interactions that occur in ‘real-world’ soil ecosystems^52,53^.

Our findings suggest that the bulk of research on soil biodiversity and AG-BG linkages is conducted in temperate biomes such as broadleaf mixed forest^5,13,14^. Notably absent are predator–prey trophic linkages drawn from biodiversity hotspots such as South and Southeast Asia, the Tropical Andes or the Horn of Africa. This is a critical knowledge gap, as soil organisms in these areas are likely to be diverse regulators of multiple soil functions^32,54^ which underpin primary productivity and food security in locally prevailing low-input smallholder systems. In addition to geographical limitations, many empirical studies either involve laboratory assays (53% studies) or draw upon single sampling efforts at a confined physical location, e.g., behavioral observations, web content analysis or brood cell dissections. Doing so may introduce important biases and can divorce empirical assessments from reality as typified by e.g., fine-scale heterogeneity within soil ecosystems^55^, temporal shifts in AG-BG interplay within ephemeral agroecosystems^37^, or plant-soil feedbacks and associated legacy effects^56^. Routinely, empirical assessments focus on unrealistic bi-trophic interactions within isolated ecosystem compartments^57^ and deploy popular methods, e.g., behavioral observation, (sentinel) prey removal or predator exclusion assays. Approaches such as faecal analysis (*n* = 19), crop content flushing or digestive tract dissection (*n* = 36) may yield a more complete picture of feeding history. Similarly, fine-resolution patterns or the feeding behavior of consumers with external digestion can be illuminated through either serological or molecular gut content assays, faecal pellet dissections, and next generation sequencing^16,58,59^. Pairing molecular assays with in-field manipulative trials or functional response models can unveil spatio-temporal feeding patterns of natural enemies of crop pests^60,61^ and provide a ‘reality check’ for theorists^62^. Also, network analyses and pulse-labelling with isotopic markers can help to unravel AG-BG structures and elucidate particular trophic interactions^57,63,64^.

Four substantial problems are encountered in food web analysis^52^, and those also permeate our recorded patterns. First, among the 1947 (non-unique) realized linkages, as few as 0.4% entail cannibalistic self-loops for a handful of species. Cannibalism is a common though largely neglected attribute of population regulation, is observed across feeding guilds and within soil food webs^65^ and determines predator–prey coexistence^66^. While (cannibalistic) self-looping is widespread and ecologically pertinent, 98.5% studies (99.6% links) do not take it into account. Second, the incredible diversity of soil-dwelling biota is poorly represented. Soil animals are thought to make up 23% of the diversity on Earth, with one square meter of soil often containing tens of thousands of microarthropods and hundreds to thousands of macro-invertebrates^67^. Yet, realized linkages are drawn for a mere 372 resource items, while only 0.3% out of an estimated 25,000 ant species are taken into consideration. Even within this small complement of soil-dwelling organisms, key taxa such as Entognatha, Chilopoda, Diplopoda or Malacostraca appear to be underrepresented—especially in empirical assessments (Fig. 5). Third, though any given animal is potentially fed upon by 10–1000 different consumers^52^, as little as 2.8 realized links (range 1–51) are drawn per resource item. While 11–27 resource items are recorded for natural enemies such as *G. aculeifer*, *S. scimitus* or *D. coriaria*, any given study only explored 1.7–2.0 (non-unique) links. Irrespective of the potential shortcomings in our study, most functional studies are thus reductionist and consider an absurdly low number of links. Fourth, organisms of variable size and age structure assume distinct trophic roles, but these parameters are rarely considered. As three-dimensional interconnected habitats, soil pores of varying size represent distinct spheres of influence, functional domains and niches^54,68^ and impose important constraints on predation^65,69^. Though microorganisms (< 200 μm) and both adult and immature mesofauna (100 μm–2 mm) or macro-organisms operate within such niches, adult stages of large-bodied, epigeic top predators and target prey (e.g., crop pests) feature disproportionately in realized links. Mid-size opportunistic feeders and omnivores are prone to be overlooked^53,70^; as such, lycosid spiders are primarily treated as top predators and collembolans as saprophages irrespective of substantial inter- and intra-specific variability in foraging mode and dietary spectrum. Considering how the trophic position of organisms increases with body size^71^, most empirical insights thus do not pertain to the small-scale, fast-revolving interlocking mechanisms within the soil biotic clockwork^8^. The above issues suggest a profoundly incomplete portrayal of the complexity and causal dynamics within multi-trophic AG-BG food webs^6^.

Soil food webs are typified by high levels of functional redundancy, which in turn shape ecosystem resilience and adaptability^49,72,73^. By visualizing trophic linkages at different taxonomic resolutions, our study facilitates trophic grouping, helps identify functional complementarities within or between individual consumer and resource guilds^1,20,46^, and guides future experimental work^2^. Also, by assigning certain species to either target or amplifiable prey groups and AG or BG realms^46^, early steps are taken towards a targeted manipulation of soil biota^42^. For example, in wheat systems of North America, an abundant complex of BG invertebrates -including amplifiable groups such as collembolans- (in-)directly engages in predation on AG herbivores^60^. Next steps can involve the addition of complementary data layers—e.g., organismal life history, r/K reproductive strategies, soil features, phytobiomes or landscape heterogeneity^37,44,68,74–76^. Subsequently, energy flux dynamics can help to anticipate how certain trophic guilds respond to e.g., species loss, microbial inoculation or carbon addition^31,49,77^. Evidently, before any credible inferences can be made regarding ecosystem service delivery, large strides need to be made in the discovery, functional characterization and manipulation of soil biodiversity^37,78^.

A science-based manipulation of trophic interactions carries major social-ecological benefits. Global warming is anticipated to disrupt the entire soil meta-organism^50,79^, but a preservation of detrital food webs and active predator communities can reduce climate change feedbacks and support belowground C storage^24,25,80^. Many predatory invertebrates prove sensitive to heating or drought^81^, with rainfall anomalies or elevated CO_2_ levels likely to cause population declines, unbalanced predator–prey ratios, trophic mismatches and a re-routing of trophic cascades^82–84^. A holistic management of soil biodiversity can mitigate some of the above threats^85^, favor agroecosystem resilience and help unlock the full potential of ecological intensification^41,42,86^. For example, invertebrate predators may mediate the outcome of plant-soil feedbacks by supporting improved plant productivity, root development or N fixation^87^. Equally, the conservation of generalist consumers can bolster plant health^33^ and curb usage of synthetic pesticides^47,88^. Agroecological outcomes can be improved through nutrient subsidies, mulching or conservation tillage tailored to specific farming systems, predator guilds or management targets^89–91^. Multi-trophic trait interactions can hereby relate predator abundance to ecosystem service delivery^92^, while a tactical pairing of soil functional ecology with other disciplines (e.g., agronomy, weed science) can help attain sustainable pest control^93–95^ and facilitate farming systems redesign^86,96^.

In conclusion, soils are self-organized ecological systems in which invertebrates are thought to take on a role as “conductors of microbial symphonies”^8,54^. However, ecologists’ depiction of (soil-borne) trophic interactions remains a caricature of real communities^52^ and considerable work remains in order to gain far more robust, realistic insights. This ‘knowledge deficit’ prevents scientists from steering the delivery of ecosystem services (e.g., biological control), and hampers progress in climate change mitigation, biodiversity preservation and agroecological intensification. As the immense pool of biodiversity within global soils can provide vital insurance against Anthropocene upsets and disturbances^26^; applied ecologists and geoscientists do well to get down to earth and systematically discover, describe and manipulate soil biota.

## Materials and methods

Our assessment of the extent of global scientific attention to soil-borne invertebrate fauna and their associated ecosystem functions (i.e., predation) and ecosystem services (i.e., biological control) was conducted in a stepwise manner (Supplementary Fig. 1). First, we queried the Web of Science Core Collection database (1900–2020) between May 15 and July 31, 2020. Boolean search strings were defined by the authors, constituting of a baseline string ‘TS = (soil AND (predate* OR prey))’ complemented with individual search terms that specifically referred to any of 36 different taxa, focused mainly on macrofauna phyla (i.e., invertebrates > 2 mm in size), while also including mites and collembola (Supplementary Table 1). The main goal of this exploratory literature search was to build a baseline for further analysis—comprising trophic linkages that departed from this initial set of 36 common taxa (i.e., in the capacity of either predator or prey items). As such, a non-exhaustive list of macro-, meso- and micro-fauna was compiled which did not necessarily include all common soil fauna (e.g., Amphipoda). For example, with the string ‘TS = (soil AND (predate* OR prey) AND (Dermaptera* OR earwig*))’, we captured literature records in which soil-dwelling earwigs either acted as predators or prey items and logged the associated resource or consumer organisms for each linkage (e.g., other invertebrates or vertebrates such as frogs, birds and lizards). For generalist (i.e., polyphagous) predators that foraged within/on soil substrates, we logged all trophic linkages that were outlined in each literature record (i.e., involving other organisms beyond the initial set of 36 taxa). Taxa were identified either at the taxonomic hierarchy of phylum, sub-class or order and comprised a diverse set of common, globally-distributed soil-foraging biota^97^. Queries were thus specifically defined to capture studies that concurrently assessed soil habitats, predation functions and a broad suite of target biota over space and time. Doing so also yielded studies in which soil was manipulated (e.g., in potted plant trials), but where predation not necessarily occurred within or on the soil habitat. Similarly, multiple records were obtained in which only one (or none) of the organisms were typical soil meso- or macro-fauna, but where the respective trophic interactions did occur on or within soil substrates. As such, organisms were included that oviposited or overwintered in the soil (e.g., the locust *D. maroccanus* or midge *D. brassicae*), flying insects which are occasionally consumed on the soil surface (e.g., mayflies consumed by caecelians) or vertebrates that consumed different soil-dwelling biota (e.g., the rainbow fish *Poecilia reticulata* which fed upon earthworms, soil mites or amphipods). When summing results for all individual taxa-level queries, the above systematic literature screening yielded a total of 3810 publications. This equaled to 2208 unique records, with the oldest record dating from 1966.

Next, abstracts of all publications were screened to select all the studies in which consumer-resource (or predator–prey) interactions were either empirically assessed or confidently deduced through advanced methods such as stable isotope analysis, fatty acid analysis or heavy metal bio-accumulation. Also, we only included studies with well-established proxies of predation e.g., abdomen width of spiders, crop mass of ground beetles, soil mass in bird pellets or predator–prey ratios when clear reference was made to prey/predator identity. Studies where the authors were unable to convincingly ascribe predation to a given organism or to identify the exact resource item for a consumer species were omitted. Only predation on live multicellular eukaryotic organisms belonging to the Phylum Animalia or their carcasses was considered, while predation of plant parts (e.g., weed seeds) and microorganisms such as protozoa, protists, rotifers or fungi was not taken into account. Also, studies that described nematode-nematode interactions, nematode predation or entomopathogenic nematode action were not considered. Free-living nematodes in the families Steinernematidae or Heterorhabditidae regularly occur in aquatic settings, often vector pathogenic bacteria and are routinely termed pathogens instead of predators^45^. As such, a core set of publications was compiled with either realized (empirically demonstrated) or inferred trophic linkages between consumer and resource organisms at varying levels of taxonomic resolution.

Key information was extracted from each of the above publications, logging data on whether the impact on primary productivity (e.g., crop yield, foliar matter, root biomass) was assessed, whether explicit mention was made of biological control services in the publication abstract or key words, and what type of habitat (i.e., agricultural, natural, urban) was investigated. Also, the geographical location (i.e., study country) and type of assay (i.e., field, laboratory, greenhouse) was recorded. We further collated details regarding the exact techniques that were employed to either empirically demonstrate or infer trophic linkages.

For the sub-set of studies that covered realized trophic linkages, the exact identity of consumer (i.e., predator) and resource (i.e., prey) organisms was recorded at the lowest possible taxonomic hierarchy and inter-organismal links were logged. Studies often investigated multi-species predator and/or prey complexes, for which each of the trophic linkages was separately considered. As such, one single study regularly yielded multiple predator–prey linkages. For resource organisms, we recorded their association with above- or belowground ecosystem compartments and assessed whether they constituted target prey (i.e., herbivores, nuisance species and crop pests) or non-target prey that potentially could be amplified through organic amendments or nutrient pulses (i.e., amplifiable prey^46^). For consumer organisms, we noted their main foraging habits as either soil-dwellers or foliage foragers while recognizing that certain taxa act across ecosystem compartments (e.g., plant-climbing by ground beetles). Lastly, for organisms in which individual life stages occupy different ecosystem compartments (e.g., the foliage feeder *Frankliniella occidentalis* Pergande pupating in the soil), we either logged the ecosystem compartment in which a given consumer-resource trophic interaction ensued or in which the resource organism (as prey target) engages in herbivory depending upon the analysis. Across the entire set of literature records, we then assessed the total number of studies, total number of linkages and average number of linkages per study for each (consumer, resource) organism identified at the species or genus level. Linkages were drawn per study and subsequently summed across studies, thus yielding a total number of (non-unique) links per organism. Indeed, a given predator–prey linkage was occasionally reported in more than one scientific study. For the purposes of data visualization, trophic linkages were equally computed at a coarser taxonomic hierarchy such as class, order or family.

For the subset of studies with inferred trophic linkages, we logged the exact identity of organisms at the lowest possible taxonomic hierarchy and assigned those individually to an upper (i.e., inferred predator) or lower (i.e., inferred prey) trophic level. Studies involving metal bio-accumulation routinely do not report concordance between a species’ trophic position and its corporal metal concentration^98^, though may reveal certain feeding links. Also, for most studies that employed stable isotope or fatty acid (FA) analysis, the contrasting isotope signatures and FA profiles of organisms could not be used to ascertain prey identity and thus imperfectly capture food web interactions^99^. Organisms were assigned to a given trophic level either as specified by the original study authors or as approximated from the isotope biplot^100^. Trophic differentiation was done by assessing the δ^15^N and δ^13^C isotopic distance between individual species and drawing trophic niches accordingly. Considering how soil food webs comprise more than two distinct trophic levels, niches of high-rank consumer taxa tend to overlap and their feeding strategies are often diffuse, we solely logged data for the highest trophic level (i.e., top predators) and the one that immediately succeeded this. Hence, for those studies in which multiple trophic levels were delineated, our approach likely obscured the relative contribution of primary decomposers (i.e., those feeding on fresh plant material and soil organic matter), while inflating the role of high-rank taxa in local trophic interactions. Conversely, our emphasis on the two highest trophic levels allowed for an accurate assignation of top predators and avoided mis-representing taxa with unclear or opportunistic feeding strategies e.g., those belonging to broad niches comprising secondary decomposers, scavengers, and mid-rank predators. The above exercise solely involved a binary approach; no effort was made to assign strength of an (inferred or realized) association between predator and prey items e.g., based upon the amount of prey consumed. Next, over the entire set of literature records, we visualized the extent to which organisms within a given taxon are distributed across (upper, lower) trophic levels. The chord diagram (Fig. 2) demonstrated the interactions of the predator–prey food web and was visualized by “circlize” package of R 4.0.2 software.

Finally, we plotted the degree of taxonomic mismatch between the sub-set of studies that inferred trophic linkages versus the one that reported realized linkages. For organisms belonging to 24 different classes, we contrasted the proportion of scientific studies that inferred their presence within upper or lower trophic levels with their relative contribution to actual realized trophic linkages (as either consumer or resource items).

## Supplementary Information

Supplementary Information.

## Data Availability

All data underlying the analyses are available at 10.25675/10217/234060.
