# Supplementary material for: Artefactual depiction of predator–prey trophic linkages in global soils
Source: Sci Rep. 2021 Dec 13;11:23861. doi: 10.1038/s41598-021-03234-7 (PMC8668944; doi:10.1038/s41598-021-03234-7)
Supplement: Supplementary file 1 — Supplementary Information. [file 41598_2021_3234_MOESM1_ESM.docx]

**Supplementary Figure 1**. PRISMA flowchart clarifying the overall study methodology, the underlying basis for selection of the two sub-set of publications (i.e., demonstrated trophic links, inferred links) and the subsequent data extraction steps.

## Exploratory literature screening

Full listing of scientific studies (over a 1900-2020 timespan) that covered any trophic interaction on / within soil media involving 36 common (macro-, meso-) fauna (n= 2,208 unique records)

Logging of respective consumer and resource organisms within each scientific publication, irrespective of the study methodology

(n= 2,208 unique records)

## Identification of consumer x resource organisms

*Demonstrated links*: Sub-set of studies in which trophic links were empirically revealed

(n= 495 records)

*Inferred links*: Sub-set of studies in which trophic links were deduced

(n= 70 records)

## Database split based upon methodological approach

Distinction of target vs. amplifiable prey, exact inter-organismal links, habitat associations

Extraction of detailed information on methodological approach, focal crop/habitat/geography, identity of consumer / resource organisms at a fine taxonomic grain, trophic level

## Data extraction & linkage description

**
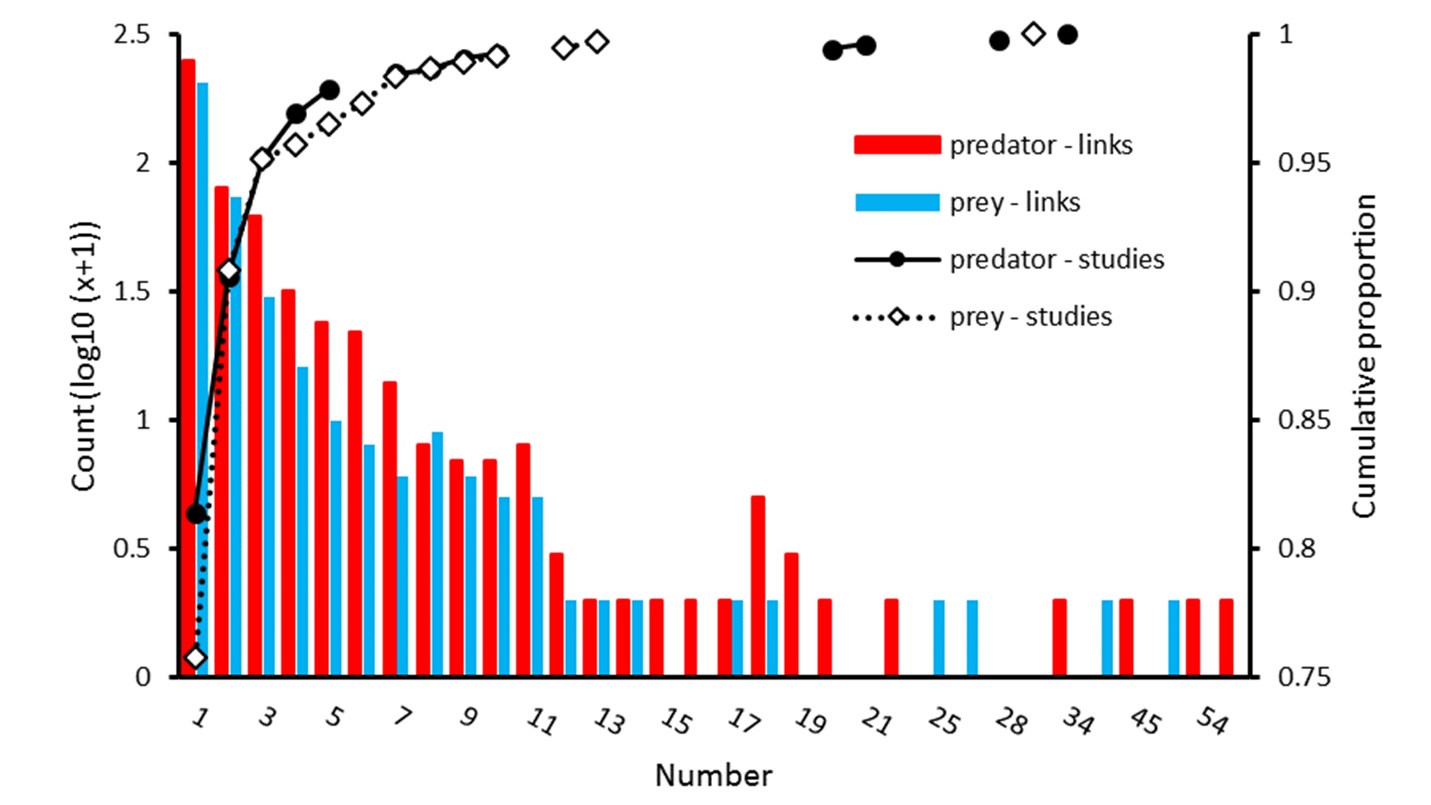
**

**Supplementary Figure 2.** Frequency distribution of the investigated consumer (i.e., predator) and resource (i.e., prey) species in terms of the number of realized trophic linkages or amount of scientific attention. The histogram plots species counts (log10x; primary Y-axis) against varying numbers of (non-unique) trophic linkages. Line charts show the cumulative proportion of predator or prey species (secondary Y-axis) that is covered in different numbers of scientific studies. X-axis either represents the number of realized trophic linkages or scientific studies. Only empirical assessments of trophic linkages are considered (*n*= 495 studies). Predator- or prey-related studies are either shown with red bars, full black dots and full black lines or blue bars, open black diamonds and dotted lines, respectively.

**
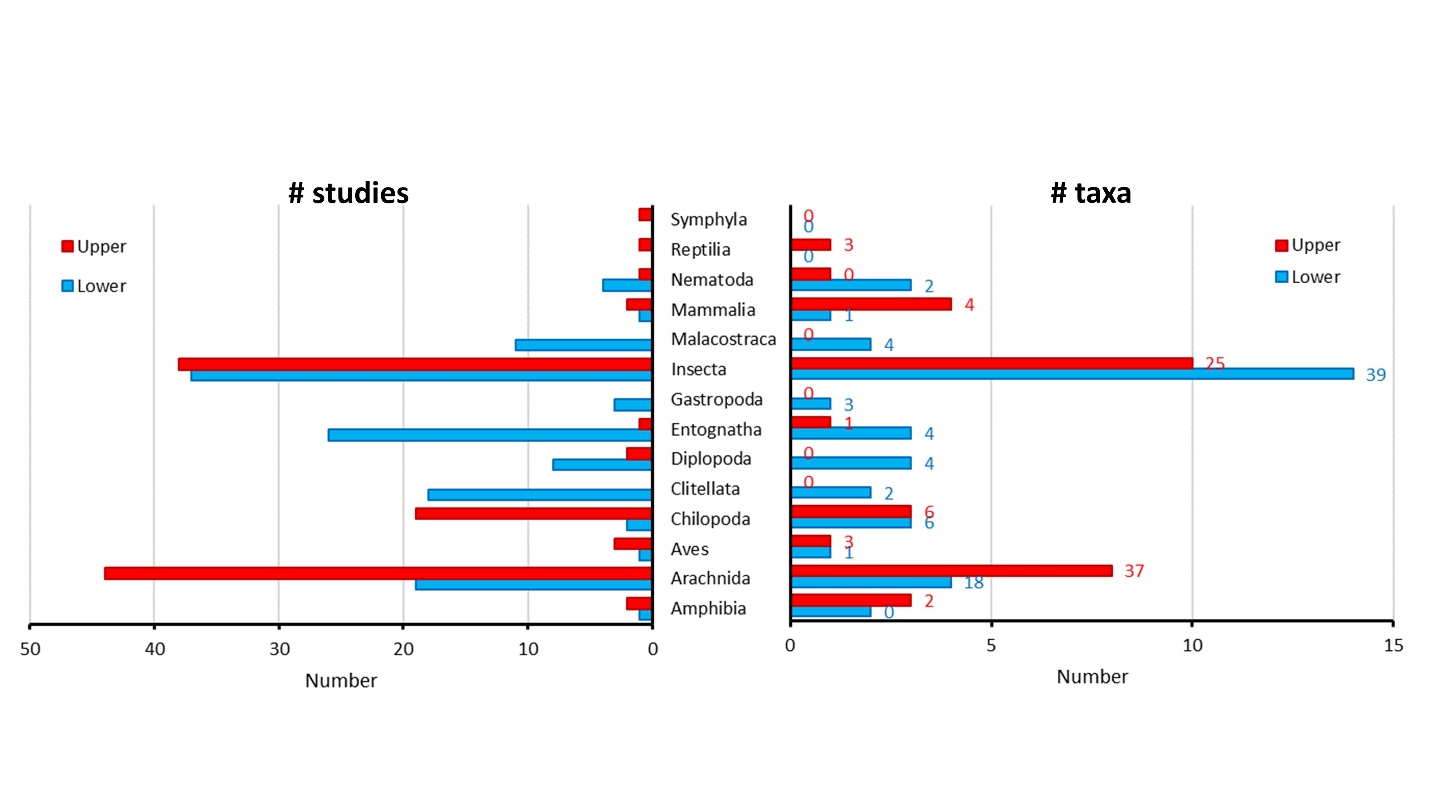
**

**Supplementary Figure 3.** Relative coverage of different classes of soil-dwelling biota in studies that infer trophic linkages (*n*= 70). Left panel: for each taxonomic group, the number of studies is shown that lists their presence in either upper (i.e., inferred consumers or predators) or lower (i.e., inferred resource or prey items) trophic levels. Right panel: for each taxonomic group, the number of orders present within either the upper or lower trophic level is depicted. Next to each bar, the number of families within a given taxonomic group is also shown.

**Supplementary Table 1**. Listing of taxa included in the initial (broad) literature search. Searches were performed by complementing a baseline Boolean search string ‘TS= (soil AND (predat* OR prey))’ with search terms that specifically referred to a target taxon, e.g., ‘Dermaptera* OR earwig*’. From the total number of logged literature records, a sub-set was subsequently retained that covered (empirically proven or inferred) predator-prey interactions with a respective taxon. Taxa mainly covered macrofauna (i.e., invertebrates > 2 mm in size), while also including mites, collembola and common soil-dwelling phyla such as ringed worms and water bears (Swift et al., 1979). Certain taxa such as terrestrial Amphipoda (Talitridae) were absent in this exploratory literature screening.

| Phylum | Subphylum | Class | Subclass/Order | Logged | Retained | Search term |
| --- | --- | --- | --- | --- | --- | --- |
| Arthropoda | Hexapoda | Entognatha | Collembola | 361 | 80 | Collembola* |
|  |  |  | Protura | 4 | 1 | Protura* |
|  |  |  | Diplura | 5 | 0 | Diplura* |
|  |  | Insecta | Blattodea | 2 | 0 | Blattodea* |
|  |  |  | Coleoptera | 623 | 150 | Coleoptera* |
|  |  |  | Dermaptera | 33 | 6 | Dermaptera* OR earwig* |
|  |  |  | Diptera | 225 | 60 | Diptera* |
|  |  |  | Ephemeroptera | 9 | 4 | Ephemeroptera* OR mayfl* |
|  |  |  | Hemiptera | 110 | 17 | Hemiptera* |
|  |  |  | Hymenoptera | 321 | 56 | Hymenoptera* |
|  |  |  | Isoptera | 107 | 12 | Isoptera* OR termite* |
|  |  |  | Lepidoptera | 203 | 16 | Lepidoptera* |
|  |  |  | Mantodea | 1 | 0 | Mantodea* |
|  |  |  | Mecoptera | 2 | 0 | Mecoptera* |
|  |  |  | Megaloptera | 1 | 0 | Megaloptera* |
|  |  |  | Neuroptera | 50 | 7 | Neuroptera* |
|  |  |  | Odonata | 6 | 0 | Odonat* |
|  |  |  | Orthoptera | 38 | 3 | Orthoptera* |
|  |  |  | Plecoptera | 4 | 0 | Plecoptera* |
|  |  |  | Pscocoptera | 8 | 0 | Pscocoptera* |
|  |  |  | Raphidioptera | 1 | 0 | Raphidioptera* |
|  |  |  | Siphonaptera | 1 | 0 | Siphonaptera* |
|  |  |  | Thysanoptera | 51 | 16 | Thysanoptera* |
|  |  |  | Trichoptera | 9 | 0 | Trichoptera* |
|  | Chelicerata | Arachnida | Acari | 579 | 63 | Acari OR mite* |
|  |  |  | Araneae | 467 | 39 | Aranea* OR spider* |
|  |  |  | Opiliones | 20 | 1 | Opilion* |
|  |  |  | Pseudoscorpion | 11 | 2 | Pseudoscorpion* |
|  |  |  | Scorpiones | 20 | 1 | Scorpion* |
|  |  |  | Solifugae | 1 | 0 | Solifuga* |
|  | Myriapoda | Chilopoda | - | 75 | 5 | Chilopod* OR centiped* |
|  |  | Diplopoda | - | 45 | 1 | Diplopod* OR milliped* |
|  | Crustacea | Malacostraca | Isopoda | 47 | 4 | Isopoda* |
| Annelida |  | Clitellata | Oligochaeta | 344 | 35 | Oligochaeta* OR earthworm* |
|  |  |  | Hirudinea | 1 | 0 | Hirudinea* |
| Tardigrada | - | - | - | 25 | 3 | Tardigrad* |
| **Total number of literature records** | | | | **3810** | **582** |  |
| **Unique literature records** | | | | **2208** | **565** |  |
